# The High Place Phenomenon: Associations With Markers of Positive and Negative Mental Health in Individuals Suffering From Specific Phobia or Agoraphobia

**DOI:** 10.1002/cpp.70038

**Published:** 2025-01-27

**Authors:** Lara Wiesmann, André Wannemüller, Tobias Teismann

**Affiliations:** ^1^ Mental Health Research and Treatment Center Ruhr‐Universität Bochum Bochum Germany

**Keywords:** anxiety sensitivity, high place phenomenon, personality traits, suicidal ideation

## Abstract

**Background:**

The Call of the Void phenomenon describes an inexplicable urge to consider dangerous or self‐destructive actions in certain situations. Previous studies have focused on the high place phenomenon (HPP), which is the sudden urge to jump from high places. One aim of this study is to replicate the previously found associations of HPP with suicidality and anxiety in a larger sample of patients suffering from flight phobia or agoraphobia. Furthermore, the influence of personality traits and protective factors, such as self‐efficacy and self‐esteem, will be examined to identify associations between the HPP and potential markers of both positive and negative mental health.

**Methods:**

The study sample comprised 612 patients (76% female; *M*_age_ = 43.77, *SD*_age_ = 12.82) suffering from clinically relevant fear of flying. Participants filled out questionnaires on experiences with the high place phenomenon, depression, personality traits, anxiety sensitivity, suicidal ideation, insecurity in social contact, flight phobia symptoms, positive mental health, self‐efficacy expectations, self‐esteem and satisfaction with life.

**Results:**

Consistent with previous findings, the high place phenomenon was known to nearly 43% of the patient sample. Multiple regression analysis showed that openness to experiences, neuroticism, suicidal ideation and insecurity in social contacts were positively related to the high place phenomenon, whereas agreeableness, self‐efficacy and self‐esteem were negatively related.

**Conclusion:**

The high place phenomenon is a common experience in individuals, whether or not they suffer from suicidal ideation. It is therefore cautioned not to interpret such experiences as an expression of a hidden death wish. Nevertheless, the occurrence of the HPP is influenced by the presence of negative and positive mental health markers. Future studies should examine the association between HPP and intrusions in the context of obsessive‐compulsive disorders.

**Abbreviations**
HPPI: High Place Phenomenon Index
Neo‐FFI: NEO‐Five‐Factors‐Inventory
Negative MH: negative mental health markers
DASS: Depression Anxiety Stress Scale
ASI: Anxiety Sensitivity Index
OASIS: Overall Anxiety Severity and Impairment Scale
FFS: Fear of Flying Scale
SIBS: Suicide Ideation and Behavior Scale
Positive MH: positive mental health markers
PHM: Positive Mental Health Scale
SWE: general self‐efficacy expectations
RSES: Rosenberg Self‐Esteem Scale
SWLS: Satisfaction With Life Scale

**Summary**

- The high place phenomenon (HPP) is a common experience in patients suffering from flight phobia or agoraphobia, reported by 43% of the present sample.
- Since HPP is common overall, it should not be considered indicative of suicidal behaviour despite a positive correlation.
- Openness to experiences, neuroticism and insecurity in social contacts are positively correlated with the HPP, while agreeableness, self‐efficacy and self‐esteem are negatively correlated.
- Future research should examine the relationship between HPP and other mental disorders, such as obsessive‐compulsive disorder, to better understand its impact on mental health.

## 1 Background

The Call of the Void phenomenon (also known as ‘L'Appel du Vide’ in French) is a popular psychological concept that describes a seemingly inexplicable tendency to consider dangerous or self‐destructive actions in certain situations. Many people have experienced the sudden urge to jump when standing on a high place, such as a bridge or viewing platform. It has been speculated to be associated with suicidal ideation or a hidden death wish. Despite being a familiar experience for many, there is relatively little research on the subject. The term *high place phenomenon* (HPP) was coined by Hames et al. (2012), who were the first to investigate the concept. Overall, HPP was only investigated in two studies (Hames et al. 2012; Teismann et al. 2020). In an American sample, Hames et al. (2012) found that 50% of participants who never considered suicide in their lives experienced aspects of the phenomenon at least once in their lives. In the group of lifetime suicidal ideators, more than 75% of the sample reported experiencing the urge to jump from a window of a tall building or from a bridge. Teismann et al. (2020) showed that 60% of a German online sample and 46% of a German patient sample were familiar with experiencing the HPP. In line with previous findings, the HPP was reported more frequently in the group of lifetime suicidal ideators (78.5%), but was still known to 45% of lifetime non‐ideators. In their studies, Hames et al. (2012) and Teismann et al. (2020) found associations of the HPP with depression and anxiety sensitivity. Anxiety sensitivity is a tendency to be fearful of arousal‐related bodily sensations and anxiety‐related symptoms (Reiss et al. 1986). Based on these findings, Hames et al. (2012) interpreted the HPP as the result of a misinterpreted safety signal in a potentially dangerous situation (‘Back up, you might fall’). However, the association between the HPP and depression, anxiety sensitivity and suicidal ideation could not be found in the German patient sample (Teismann et al. 2020). In their study, Teismann et al. compare the HPP to intrusive thoughts: Intrusive thoughts are unpleasant, fleeting and egodystonic thoughts or images of, for example, sexual, violent and blasphemous content and are often associated with anxiety or distress (Berry and Laskey 2012).

In summary, studies have shown that the high place phenomenon is a common experience. There were associations with negative mental health markers such as depression and anxiety sensitivity. Although experiences of the high place phenomenon are strongly associated with current and lifetime suicidal ideation (Hames et al. 2012; Teismann et al. 2020), the studies showed that the experience also occurred in participants who have never suffered from suicidal ideation and is therefore not in itself a sign of psychopathology or a hidden death wish.

Overall, there is too little research to fully understand the high place phenomenon. The aim of this study is, therefore, to characterize the HPP more broadly, especially since the patient sample used in the study by Teismann et al. (2020) was comparatively small (*n* = 94). This study examines associations between the HPP and anxiety sensitivity, anxiety severity and impairment, fear of flying, depression, stress and suicidality in a much larger sample of patients suffering from both specific phobia and agoraphobia.

In order to gain a better understanding of the high place phenomenon and its occurrence, it is necessary to investigate the relationship with other psychological constructs as well. Therefore, this study analyses the relationship with personality traits, that is, the Big Five personality traits extraversion, neuroticism, openness to experience, agreeableness, conscientiousness and potential protective factors, that is, emotional and psychological well‐being, general self‐efficacy expectations, self‐esteem and life satisfaction (Brailovskaia et al. 2019; Ferring and Filipp 1996; Franke 2012; Gurung et al. 2019; Nikčević et al. 2021; Schwarzer and Warner 2013).

## 2 Methods

### 2.1 Participants

The sample comprises of *N* = 612 participants (75.65% female; *M*_age_ = 43.77, *SD*_age_ = 12.82, range: 18–78 years) suffering from a specific phobia (fear of flying; 75.65%), agoraphobia (11.60% agoraphobia with and 11.60% agoraphobia without panic disorder), panic disorder (0.49%), generalized anxiety disorder (0.16%), social phobia (0.16%), major depression (0.16%) or obsessive‐compulsive disorder (0.16%). All patients were diagnosed by licensed psychotherapists or post‐graduate clinical psychologists using the Short Interview for Mental Disorders (Margraf and Cwik 2017). Among the participants, 16.67% reported being in psychological treatment, while 51.63% indicated that they had received treatment in the past. In terms of their job, most people work as employees (65.52%); students and trainees make up 8.66% of the sample, 6.70% are civil servants and 3.43% work as freelancers, 1.14% are job seekers, 2.61% are housewives/housemen, 1.31% are on parental leave and 10.62% have another job. More than half of the patients (53.59%) have children of their own.

### 2.2 Procedure

The patient sample comprised individuals who took part in a training program to reduce fear of flying. Wannemüller et al. (2024, manuscript in preparation) conducted this large‐group one‐session treatment at the Airport of Düsseldorf in Germany on 2 March 2024. People with a subjective high level of fear were recruited through a local radio campaign, newspaper advertisements and a report on a local television station. A website was also set up for the project, providing information about previous trials, the study team and procedures. Patients could register by e‐mail and were then allocated to the pre‐treatment psychotherapy consultation. Inclusion criteria were a minimum age of 18 years and clinically relevant fear of flying. Exclusion criteria were the presence of manic or hypomanic episodes, schizophrenia and severe post‐traumatic stress disorder or serious physical illnesses that limited flying (pregnancy, severe infectious diseases, acute otitis media, cardiopulmonary diseases, epilepsy, previous stroke and severe physical or mental disability).

Prior to the start of the actual treatment, a pre‐treatment assessment was conducted in which participants were asked to complete questionnaires about their subjective fear of flying and symptom distress at the initial assessment. Participants also answered various questionnaires including questionnaires on experiences with the high place phenomenon, personality traits, self‐esteem, self‐efficiency expectations, positive mental health, anxiety, depression and suicidal ideation, which are analysed in this study. Anxiety sensitivity was measured on the day of the actual treatment with *N* = 539. Before participation, all patients were informed about the voluntary nature of their participation, data storage and backup, and the purpose of the study. The study was approved by Ethics Committee of the Ruhr‐Universität Bochum (Ethics vote 873).

### 2.3 Measures

#### 2.3.1 High Place Phenomenon Index (HPPI; Hames et al. 2012)

The HPPI consists of three items that assess the frequency of lifetime experiences with the high place phenomenon using a 6‐point Likert type scale ranging from (0) never to (5) always: Item 1: *When standing on the edge of a tall building or walking on a bridge, have you ever had the urge to jump?* Item 2: *When you see a tall building or are walking on a bridge, have you ever thought about what it would be like to jump off it?* Item 3: *When you are inside a tall building have you ever imagined jumping out a window?* The German version of the HPPI was developed using a translation‐back‐translation process in accordance with relevant guidelines for the translation of psychometric instruments (Teismann et al. 2020). The original scale and the German HPPI showed good internal consistency (*α* = 0.84 to 0.86; Hames et al. 2012; Teismann et al. 2020) whereas the internal consistency in this sample was acceptable (*α* = 0.79).

#### 2.3.2 NEO‐Five‐Factors‐Inventory (NEO‐FFI; Borkenau and Ostendorf 2008)

The NEO‐FFI consists of 60 items and measures the personality factors Neuroticism, Extraversion, Openness to Experience, Agreeableness and Conscientiousness on a 5‐point Likert scale. The internal consistency in the previous samples was on average acceptable (α = 0.78; Schmitz et al. 2001); in this sample, it showed an internal consistency of α = 0.75 (internal consistencies of the subscale Neuroticism α = 0.85, Extraversion α = 0.82, Openness to Experience α = 0.71, Agreeableness α = 0.77 and Conscientiousness α = 0.85).

#### 2.3.3 Depression Anxiety Stress Scales (DASS; Lovibond and Lovibond 1995)

The DASS consists of 21‐items and measures depressive mood, anxiety and stress during the past week (DASS‐Depression‐Scale, DASS‐Anxiety‐Scale, DASS‐Stress‐Scale). The items are rated on a 4‐point (0–3) Likert scale. The Internal consistency for the DASS in total was good in a range of *α* = 0.88–0.96 (Brown et al. 1997). The internal consistency of the scale and its subscales was excellent in this sample (DASS: *α* = 0.94, Subscales Depression α = 0.85, Anxiety α = 0.85 and Stress α = 0.89).

#### 2.3.4 Anxiety Sensitivity Index (ASI; Reiss et al. 1986)

The ASI consists of 16 Items and measures the extent to which individuals are concerned about the potential negative consequences of experiencing anxiety symptoms. The items are rated on a 5‐point Likert. The scale has been found to have strong internal consistency and test–retest reliability in previous samples (e.g., Taylor 1999). Internal consistency in this sample was excellent with *α* = 0.99.

#### 2.3.5 Overall Anxiety Severity and Impairment Scale (OASIS; Norman et al. 2006)

The Overall Anxiety Severity and Impairment Scale is a 5‐item self‐report measure of impairment associated with any anxiety disorder. On a 5‐point Likert scale, it assesses frequency of anxiety, intensity of anxiety symptoms, behavioural avoidance and functional impairment associated with anxiety. Test–retest reliability, convergent and discriminant validity were excellent in a sample of university students (Norman et al. 2006) and items had a high degree of internal consistency. In this sample the internal consistency was good with *α* = 0.83.

#### 2.3.6 Fear of Flying Scale (FFS; Haug et al. 1987; Mühlberger and Pauli 2011)

The German version of the Fear of Flying Scale consists of 21 items that can be assigned to an overall scale and five subscales (generalized fear of flying, anticipation, flying, turbulence, landing). Each item is rated on a 5‐point Likert scale, and the FFS has shown to have a medium to high retest reliability and a high internal consistency (Mühlberger and Pauli 2011). The internal consistency in this sample was high as well (*α* = 0.92).

#### 2.3.7 Suicide Ideation and Behavior Scale (SIBS; Teismann, Glaesmer, and Forkmann 2017)

The SIBS uses six of the nine items to assess the frequency and intensity of passive and active suicidal thoughts, suicidal intentions, suicidal impulses and suicidal plans within the last 4 weeks. The last three items measure occurrence and frequency of suicide attempts in the past 4 weeks and over the lifetime. Internal consistency was good in previous samples (*α* = 0.87, Teismann 2018). In this sample, it was acceptable with *α* = 0.75.

#### 2.3.8 Positive Mental Health Scale (PMH; Lukat et al. 2016)

The PMH‐scale consists of 9 items that assess aspects of emotional and psychological well‐being. The responses are rated on a 4‐point Likert scale. The scale showed high internal consistency, good retest‐reliability, scalar invariance across samples and over time, good convergent and discriminant validity (Lukat et al. 2016). The internal consistency in this study was very good (*α* = 0.91).

#### 2.3.9 General Self‐Efficacy Expectations (SWE; Schwarzer and Jerusalem 1995)

The German SWE is a 10‐item self‐report measure of general optimistic self‐efficacy. It measures optimistic competence expectancy, that is, confidence in being able to cope with a difficult situation in which success is attributed to one's own competence. The items are measured on a 5‐point Likert scale. In German samples, the internal consistencies have been shown to be very good (*α* = 0.80–0.90; Schwarzer and Jerusalem 1995). In this sample, internal consistency was very good as well (*α* = 0.90).

#### 2.3.10 Rosenberg Self‐Esteem Scale (RSES; Martín‐Albo et al. 2007; Rosenberg 1965)

The RSES comprises 10 statements that can be used to assess the degree of positive or negative self‐esteem. Rosenberg defines global self‐esteem as a person's positive or negative attitude towards the self. The items are rated on a 4‐point scale. Reliability, validity and internal consistence of the German version of the RESES have been found to be good (Martín‐Albo et al. 2007). In this sample, the scale showed a very good internal consistency with *α* = 0.89.

#### 2.3.11 Satisfaction With Life Scale (SWLS; Diener et al. 1985; Glaesmer et al. 2011)

The German version of the SWLS was used to measure life satisfaction. The five‐item scale is scored on a 7‐point scale and has shown very good internal consistency and also high validity (α = 0.92, Westaway and Maritz 2003). The internal consistency of the SWLS in this sample is good (α = 0.82).

### 2.4 Statistical Analyses

Statistical analyses were conducted with R Studio (version: Version 2023.12.1+402). Descriptive data and bivariate correlations of the variables were calculated. In three steps, hierarchical regressions were used to analyse the relationship between (1) personality traits, (2) negative mental health markers and (3) positive mental health markers, suicidal ideation and experiences with the high place phenomenon. Therefore, in the first analysis, personality traits (NEO_FFI) were used as independent variables. In a second regression model, possible negative markers of mental health (anxiety sensitivity [ASI], depressive symptoms [DASS‐D] and anxiety symptoms [OASIS] and flight phobia symptoms [FFS]) were used as independent variables. Finally, the predictive effect of possible positive markers of mental health (positive mental health [PHM], self‐efficacy expectations [SWE], self‐esteem [RSES] and satisfaction with life [SWLS]) on the HPP were analysed. All regression analyses were controlled for age and gender in the first step and suicidal ideation in the last step. There was no violation of the multicollinearity assumption as all values of tolerance were > 0.25, and all variance inflation factor values were < 5.

## 3 Results

### 3.1 Descriptive Statistics, Correlations and Group Differences

The high place phenomenon (HPPI) was known to *n* = 263 (43%) participants. There were no significant group differences between male (45.95% of *n* = 148) and female (41.88% of *n* = 463) participants regarding experiences with the HPP, χ ^2^ = 0.593, *df* = 1, *p* = 0.44. Furthermore, there were no significant group differences between patients suffering from agoraphobia with/without panic disorder (41.55%) or specific fear of flying (43.20%) regarding experiences with the HPP, χ ^2^ = 0.06, *df* = 1, *p* = 0.80. Forty‐seven participants (7.77%) reported to have suffered from suicidal ideation within the last 4 weeks: 85.11% of these participants reported experiences with the high place phenomenon. Suicidal ideators and non‐ideators (39.47%) did differ in the rate the HPP was familiar to them, χ ^2^ = 35.038, *df* = 1, *p* = 0.000*. Lifetime suicide attempts were reported by eight participants (1.31%). Table 1 shows descriptive statistics for each measure and correlations to the high place phenomenon. Correlation analyses indicated that all variables—except gender and anxiety sensitivity—were associated to the HPP.

**TABLE 1:** Sample, means and standard deviations of study variables.

		*n*	HPPI *m* (*SD*)
Sample		612	0.34 (0.58)
			***r***
Gender	Male	148	−0.04
	Female	463	
		*m* (*SD*)	
Age		43.77 (12.82)	−0.09*
Neo‐FFI	Extraversion	2.26 (0.58)	−0.18**
	Openness to experience	2.47 (0.52)	0.14**
	Agreeableness	2.96 (0.49)	−0.15**
	Conscientiousness	2.86 (0.57)	−0.15**
	Neuroticism	1.82 (0.67)	0.28**
Negative MH	DASS	0.56 (0.49)	0.44***
	ASI	21.59 (79.86)	0.06
	OASIS	2.34 (0.78)	0.18***
	FFS	49.79 (12.06)	0.08*
	SIBS	1.02 (0.10)	0.44***
Positive MH	PHM	1.97 (0.60)	−0.23***
	SWE	2.84 (0.47)	−0.12***
	RSES	3.30 (0.56)	−0.31***
	SWLS	2.76 (0.54)	−0.12***

Abbreviations: ASI, Anxiety Sensitivity Index; DASS, Depression Anxiety Stress Scale; FFS, Fear of Flying Scale; HPPI, High Place Phenomenon Index; m, mean; Negative MH, negative mental health markers; Neo‐FFI, NEO‐Five‐Factors‐Inventory; OASIS, Overall Anxiety Severity and Impairment Scale; PHM, Positive Mental Health Scale; Positive MH, positive mental health markers; *r*, Person correlation; RSES, Rosenberg Self‐Esteem Scale; SD, standard deviation; SIBS, Suicide Ideation and Behavior Scale; SWE, general self‐efficacy expectations; SWLS Satisfaction With Life Scale.

* *p* < 0.05.

** *p* < 0.01.

*** *p* < 0.001.

### 3.2 Hierarchical Regression Analysis

#### 3.2.1 Prediction of Experiences With the High Place Phenomenon by Personality Traits

The results of the first hierarchical regression analysis including personality variables are presented in Table 2. Besides the prediction of the HPP by suicidal ideation, experiences of the high place phenomenon were associated with openness to experience, agreeableness and neuroticism.

**TABLE 2:** Multiple linear models for the prediction of the HPPI by personality traits.

	Model 1			Model 2			Model 3		
	B	T	*p*	B	T	*p*	B	T	*p*
Age	−0.00	−2.26	0.02*	−0.00	−1.73	0.08	−0.00	−1.00	0.31
Gender	−0.05	−0.86	0.39.	−0.06	−1.13	0.25	−0.02	−0.50	0.63
Extraversion	—	—	—	−0.06	−1.28	0.20	−0.02	−0.67	0.50
Openness to experience	—	—	—	0.19	4.38	0.000***	0.16	3.84	0.000***
Agreeableness	—	—	—	−0.13	−2.63	0.00**	−0.10	−2.15	0.03*
Conscientiousness	—	—	—	−0.01	−0.23	0.81	−0.03	−0.84	0.40
Neuroticism	—	—	—	0.19	4.60	0.000***	0.11	2.96	0.003**
SIBS							2.18	10.19	0.000***
Model	Adj. *R* ^2^ = 0.006			Adj. *R* ^2^ = 0.11			Adj. *R* ^2^ = 0.241		
	*F*(2, 609) = 2.96			*F*(7, 604) = 12.1			*F*(8, 603) = 25.37		
	*p* = 0.05			*p* < 0.000			*p* < 0.000		

Abbreviations: HPPI, High Place Phenomenon Index; SIBS, Suicide Ideation and Behavior Scale.

* *p* < 0.05.

** *p* < 0.01.

*** *p* < 0.0001.

#### 3.2.2 Prediction of Experiences With the High Place Phenomenon by Negative Mental Health Markers

The results of the second hierarchical regression analysis including possible negative mental health markers (controlled for age and gender) are presented in Table 3. Depression and suicidal ideation were associated with experiences with the high place phenomenon. None of the other variables of negative mental health predicted the HPP.

**TABLE 3:** Multiple linear models for the prediction of the HPPI by negative mental health markers.

	Model 1			Model 2			Model 3		
	B	T	*p*	B	T	*p*	B	T	*p*
Age	−0.00	−2.26	0.02*	−0.00	−1.18	0.24	−0.00	−0.68	0.50
Gender	−0.05	−0.86	0.39.	−0.09	−1.51	0.13	−0.04	−0.76	0.45
DASS‐S	—	—	—	−0.08	−1.17	0.24	−0.04	−0.66	0.50
DASS‐D	—	—	—	0.29	4.56	0.000***	0.13	2.18	0.02*
DASS‐A	—	—	—	0.02	0.32	0.75	−0.00	−0.05	0.96
FFS	—	—	—	0.03	0.71	0.48	−0.00	−0.12	0.90
OASIS	—	—	—	0.06	1.72	0.09	0.06	1.88	0.06
ASI	—	—	—	0.01	1.52	0.13	0.00	1.20	0.23
SIBS	—	—	—	—	—	—	2.25	9.61	0.000***
Model	Adj. *R* ^2^ = 0.006			Adj. *R* ^2^ = 0.08			Adj. *R* ^2^ = 0.211		
	*F*(2, 609) = 2.96			*F*(8,530) = 6.462			*F*(9, 529) = 17		
	*p* = 0.05			*p* < 0.000			*p* < 0.000		

Abbreviations: ASI, Anxiety Sensitivity Index; DASS‐A, Depression Anxiety Stress Scale‐Anxiety Subscale; DASS‐D, Depression Anxiety Stress Scale–Depression Subscale; DASS‐S, Depression Anxiety Stress Scale–Stress Subscale; FFS, Fear of Flying Scale; HPPI, High Place Phenomenon Index; OASIS, Overall Anxiety Severity and Impairment Scale; SIBS, Suicide Ideation and Behavior Scale.

* *p* < 0.05.

** *p* < 0.01.

*** *p* < 0.001.

#### 3.2.3 Prediction of Experiences With the High Place Phenomenon by Positive Mental Health Markers

The results of the third hierarchical regression analysis including possible positive mental health markers (controlled for age and gender) are presented in Table 4. Experiences with the high place phenomenon were predicted by self‐esteem and suicidal ideation.

**TABLE 4:** Multiple linear models for the prediction of the HPPI by positive mental health markers.

	Model 1			Model 2			Model 3		
	B	T	*p*	B	T	*p*	B	T	*p*
Age	−0.00	−2.26	0.02*	−0.00	−1.02	0.31	−0.00	−0.57	0.57
Gender	−0.05	−0.86	0.39 .	−0.04	−0.94	0.35	−0.02	−0.46	0.65
SWLS	—	—	—	0.01	0.11	0.91	0.01	0.19	0.85
PMH	—	—	—	−0.05	−0.72	0.47	−0.05	−0.80	0.42
SWE	—	—	—	0.12	1.97	0.049*	0.09	1.60	0.10
RSES	—	—	—	−0.35	−5.76	0.000***	−0.20	−3.50	0.000***
SIBS	—	—	—	—	—	—	2.18	9.81	0.000***
Model	Adj. *R* ^2^ = 0.006			Adj. *R* ^2^ = 0.10			Adj. *R* ^2^ = 0.221		
	*F*(2, 609) = 2.96			*F*(6, 605) = 12.12			*F*(7, 604) = 25.8		
	*p* = 0.05			*p* < 0.000			*p* < 0.000		

Abbreviations: HPPI, High Place Phenomenon Index; PHM, Positive Mental Health Scale; RSES, Rosenberg Self‐Esteem Scale; SIBS, Suicide Ideation and Behavior Scale; SWE, general self‐efficacy expectations; SWLS, Satisfaction With Life Scale.

* *p *< 0.05.

** *p *< 0.01.

*** *p *< 0.001.

## 4 Discussion

The aim of the present study was to examine experiences with the high place phenomenon in a large German sample of patients suffering from specific phobia or agoraphobia with/without panic disorder. Overall, 43% of the participants reported experiences with the high place phenomenon at least once in their lifetime. This prevalence rate is in line with the results of previous studies in which the phenomenon was investigated in an American student sample (30%–53%; Hames et al. 2012), in a German online (60%) and in a German patient sample (45%; Teismann et al. 2020). Approximately 85% of individuals reporting current suicidal ideation experienced HPP in this study. Teismann et al. (2020) investigated both current and lifetime suicide ideators and found prevalence rates around 80%. This indicates that the HPP is a common phenomenon that is often reported in the context of suicidal crises. Nevertheless, 39% of the individuals who do not suffer from suicidal ideation, also reported experiences with the HPP (Hames et al. 2012; Teismann et al. 2020). Accordingly, it seems unlikely that HPP is an expression of a hidden death wish. However, as the current study did not assess lifetime suicidal ideation and since all studies so far made use of self‐report assessments more fine‐grained studies using implicit association test (Sohn et al. 2021) might be warranted to come to a more definite conclusion about association between the HPP and a hidden death wish.

For the first time, this study analysed associations between the HPP and personality factors. Hierarchical regression models were used to show that higher levels of neuroticism and openness to experience and lower levels of agreeableness predicted the occurrence of the phenomenon. The personality factor neuroticism is a well‐researched risk factor of affective disorders (e.g., Bienvenu et al. 2001; Brown, Chorpita, and Barlow 1998; Lyon et al. 2021). People who score high on neuroticism are more likely than average to experience feelings such as anxiety, irritability, depression, social anxiety, impulsivity and vulnerability (Borkenau and Ostendorf 2008). Therefore, when standing on a high platform and experiencing irritation, stress or fear, these people may be more susceptible to experiencing HPP. The personality factor openness to experience describes open‐mindedness, curiosity, tolerance, and interest in art and culture, creativity, and educational experiences (Borkenau and Ostendorf 2008). It can be speculated that individuals with high levels of curiosity and creativity may be more likely to visit elevated places, such as viewing platforms, and therefore experience HPP more frequently. But to replicate and better understand this association, further research is needed. In this study lower levels of agreeableness predicted the occurrence of the high place phenomenon. People with a lower score of agreeableness are more sceptical and cynical, have less trust in others and are less empathetic (Borkenau and Ostendorf 2008). Previous findings suggest that the ability to cooperate with others and have a friendly attitude is the reason, that a high level of the personality trait is negatively correlated to depression (He and Li 2022). It might therefore be a protective factor to psychological phenomena like the HPP as well.

Depression predicted the occurrence of HPP not only in this, but in all previous studies (Hames et al. 2012; Teismann et al. 2020). People who suffer from depression are theorized to selectively attend to negative cues in their environment and have the tendency to create more negative meanings to explain ambiguous information (Beck and Haigh 2014; Clark, Beck, and Alford 1999). This cognitive bias could explain the occurrence of the negatively valenced thought of jumping when standing on a high place in the first place. Secondly, the tendency to interpret this ambiguous thought as dangerous could lead to greater rumination, thereby increasing the likelihood of its occurrence. Moreover, a ‘sudden urge to jump’ leading to catastrophic outcomes could also represent one of several threat‐associated cognitions in height‐related situations. Others are, for example, slipping, losing control of the body or fainting in these situations and therefore falling (e.g., Steinman and Teachman 2011). People with increased suicidal ideation and depressive symptoms could be more prone to developing threat‐associated cognitions in height‐related situations that are linked to suicidal behaviour because for them this may represent the closest explanation for catastrophic outcomes in altitude situations which therefore they fear most and, in turn, have an increased likelihood to experience. It would be interesting to see to what extent, conversely, people with panic disorder are more likely to anticipate a physical loss of control in height situations. Hames et al. (2012) and Teismann et al. (2020) found evidence of associations between the HPP and anxiety sensitivity in their non‐clinical samples. As there was no evidence in the specific phobia sample, Teismann et al. (2020) concluded that the lack of evidence might be due to the fact that the specific type of a phobic disorder is less characterized by the fear of certain bodily symptoms (Teismann et al. 2020). However, the current study, which included patients suffering from agoraphobia or specific phobic, also found no association between the HPP and anxiety sensitivity suggesting that this association cannot be found in clinical samples.

This study also analysed the influence of positive mental health markers and could show a negative association between self‐esteem and the high place phenomenon, that is, individuals with higher self‐esteem are less likely to have experienced the HPP. People with a higher self‐esteem potentially experience the HPP as less threatening and therefore remember the experiences less well (potentially comparable to the mechanisms of obsessive thoughts; Shafii Kahani, Hassani, and Shakeri 2022).

The following limitations should be considered when interpreting the results of the current study. The data was collected in a Fear of Flying project, where participants were highly motivated to overcome their fear of flying. This, together with the fact that the sample consists only of Caucasians, affects the generalizability of the results to other people suffering from agoraphobia or fear of flying and limits the generalizability to the population. Second, the percentage of people with current suicidal ideation (47 participants, i.e., 7.77% of total) is very small compared to the sample size. To discuss the relationship between suicidality and HPP, the group size should be larger, and the survey should include lifetime suicidal ideation. Third, the cross‐sectional research design limited the interpretation of the temporal and causal relationship between the study variables. And last, depression symptoms were only assessed with the depression scale of the DASS. Future studies should use specific scales for depression symptoms, such as the BDI (Hautzinger, Keller, and Kühner 2006).

In summary, this study lays the foundation for future studies. After characterizing the HPP and investigating its relationships with other psychological constructs, future studies should focus on more specific questions. They should examine the hypotheses arising from these findings about the origin of HPP by using other methods than self‐report, like implicit association tests (Sohn et al. 2021), to test, whether there are connections to an implicit death wish. Furthermore, VR glasses could also be used to provoke the HPP in the laboratory and capture previous and emerging thoughts and feelings as well as the following interpretation of the high place phenomenon. Further research should also investigate whether this or similar phenomena (e.g., the call of the void) occur in situations other than high places and asses the relation to obsessive thoughts and intrusions.

## 5 Conclusion

The current study replicates previous findings that the high place phenomenon is a common experience that is known to both individuals with suicidal ideation and individuals without suicidal ideation. The phenomenon can be associated with specific personality factors and is positively correlated with negative mental health markers and negatively correlated with positive mental health markers.

## Author Contributions

T.T. and A.W. conceived and designed the study. A.W. and T.T. recruited the data. L.W. analysed the data and wrote the paper together with T.T.

## Ethics Statement

All participants provided written informed consent. Furthermore, all procedures performed in studies involving human participants were in accordance with the ethical standards of the institutional and/or national research committee and with the 1964 Helsinki declaration and its later amendments or comparable ethical standards. The studies were approved by the responsible Ethics Committee of the Faculty of Psychology, Ruhr‐Universität Bochum.

## Consent

All participants provided consent to publish all anonymized data reported in this publication.

## Conflicts of Interest

The authors declare no conflicts of interest.

## Data Availability

All relevant data are reported within the paper. Analysed data are available from the corresponding author on reasonable request.
